# Raman Spectroscopy-Based Assessment of the Liquid Water Content in Snow

**DOI:** 10.3390/molecules27030626

**Published:** 2022-01-19

**Authors:** Ettore Maggiore, Matteo Tommasini, Paolo Maria Ossi

**Affiliations:** 1 Dipartimento di Chimica, Materiali e Ingegneria Chimica “G. Natta”, Politecnico di Milano, 20133 Milano, Italy; ettore.maggiore@polimi.it (E.M.); matteo.tommasini@polimi.it (M.T.); 2 Dipartimento di Energia, Politecnico di Milano, 20133 Milano, Italy

**Keywords:** snow, water, Raman spectroscopy, OH-stretching band, liquid water content

## Abstract

In snow, water coexists in solid, liquid and vapor states. The relative abundance of the three phases drives snow grain metamorphism and affects the physical properties of the snowpack. Knowledge of the content of the liquid phase in snow is critical to estimate the snowmelt runoff and to forecast the release of wet avalanches. Liquid water does not spread homogeneously through a snowpack because different snow layers have different permeabilities; therefore, it is important to track sudden changes in the amount of liquid water within a specific layer. We reproduced water percolation in the laboratory, and used Raman spectroscopy to detect the presence of the liquid phase in controlled snow samples. We performed experiments on both fine- and coarse-grained snow. The obtained snow spectra are well fitted by a linear combination of the spectra typical of liquid water and ice. We progressively charged snow with liquid water from dry snow up to soaked snow. As a result, we exploited continuous, qualitative monitoring of the evolution of the liquid water content as reflected by the fitting coefficient c.

## 1. Introduction

Snow is a granular, porous material made of a mixture of ice crystals with a variety of forms, a fraction of liquid water and water vapor in thermodynamic equilibrium [1]. The initial shape of the crystallites formed in a cloud include plates, needles, hollow columns, dendrites, depending on the combinations of temperature and water vapor supersaturation values they experience along their free fall down to the ground, where they progressively accumulate [2]. After its deposition, natural snow undergoes extensive metamorphism [3] that consists of grain sintering under mechanical compression and thermal gradients through the snowpack thickness due to mass and thermal energy fluxes, driven by the weather conditions it experiences. Sun irradiation, temperature gradients, humidity and wind are the leading factors that concur with the evolution of the layers that progressively accumulate upon successive snowfalls and build up the snowpack. The size and shape of the constituting snow grains concurrently evolve. In the laboratory, strict control of ambient temperature and supersaturation allows us to produce natural-like snow crystals with a degree of perfection higher than that of spontaneously grown crystals [4].

A relevant property of a snowpack is the liquid water content θ_w_. This results mostly when melted snow or rainwater infiltrates into the snow, changing its wetness [5]; alternatively, the presence of thermal gradients through the snowpack may drive changes of θ_w_ in snow layers at specific depths [6]. As such, θ_w_ in snow is a marker of snowmelt and snow mechanical stability [7]. Accelerated melting under strong Sun irradiation, as well as heavy rain on snow, lead to increased θ_w_ values and can result in a flood, possibly associated with severe runoff [8] or wet avalanche release. This can be full depth when water reaches the ground behind the snow cover, or, since the shear strength of snow reduces exponentially with increasing the volumetric water content [9], when a wet, buried snow layer becomes the preferential sliding surface of the avalanche. The fate of the snowpack is further affected by the lowered surface albedo of snow progressively impregnated with water [10] since considerable liquid phase amounts favor the formation of ice clusters [11] that act similar to big grains, being more efficient than small crystallites in absorbing light. Indeed, albedo is higher in snow with smaller grains and vice versa [12].

Measuring θ_w_ is admittedly difficult and demanding [13,14]. To take into account the space–time evolution of meltwater outflow that is correlated with the snowpack stability, continuous recording of θ_w_ is required. Indeed, associated with θ_w_ changes are sudden, non-linear alterations of snowpack properties and of the outflow of meltwater. Several in-situ, more or less invasive techniques to measure θ_w_ were reviewed in the past and include centrifugal, dielectric, calorimetric or dilution approaches [15,16]. The main drawbacks of such techniques are the large amount of snow and the long time necessary to perform a measurement. Thus, no successive tests can be made at a given site since the snowpack is irreversibly altered at every measurement. More gentle, non-destructive methods are based on the changes of spectral reflectance in the NIR region (920–1650 nm) of snow bearing different water contents [17,18] and ground-penetrating radar, by which an electromagnetic signal is generated and the reflected wavefield is measured, from which it is possible to map the values of θ_w_ in the sampled region. Recent satellite and ground-based remote sensing are based on the analysis of microwave radiation reflected at the Earth’s surface in comparison to a signal directly received at an antenna above the ground [19,20]. These methods leave the sampled area intact, but since the satellite repetition time is of the order of days, snow parameters can be checked only intermittently. Mountain areas are challenging for passive microwave systems, mostly due to the coarse spatial resolution, in the range of a few tens of km^2^, and to the complicated terrain topography that produces shadowing and foreshortening effects. Thus, presently, satellite-based sensing of snow properties is mostly devoted to flat areas [21].

Here we describe an innovative approach based on Raman spectroscopy for the insitu assessment of the liquid phase content in snow. We conceived this idea from the observations that first, snow consists of frozen water; second, we are concerned with discriminating among snow with different contents of liquid-phase water; and third, in the region of the OH-stretching band, the Raman spectrum (RS) of ice qualitatively differs from that of liquid water. Notably, very recently, we found a single old report (in Russian) where Raman spectroscopy was proposed as a conceptual tool to determine the θ_w_ of snow [22]. Other optical spectroscopy techniques, such as reflectance spectroscopy, were used to investigate snow metamorphism [23] and to measure snow albedo to derive from it the snow grain size, also taking into account the presence of different concentrations of dust contaminants [24].

In our study, we used two reference types of snow with markedly different average grain sizes (0.2 mm; 1.6 mm). By this choice, we expect that the infiltration strategy of equal, controlled amounts of water, homogeneously deposited on the sample surface, differs in the two snow kinds. We recorded RS from the snow samples at a fixed height below the water-wet surface, at fixed delays with respect to the wetting time. We observed that the RS of snow with different fractional contents of the liquid phase, driven by the degree of water infiltration, can be described well by a weighted linear combination of the spectra of ice and liquid water. For both kinds of tested snow, we plotted vs. time the weighting factor (*c*) as well as the independently measured liquid phase fraction. We found a smooth behavior of *c* with two evident thresholds. Each threshold occurs at the same value of liquid phase fraction for both kinds of snow. By our method, we collected RS from specific points in the snow volume. Even though punctual information could be of less interest when the global liquid water content in a snow volume is required, local Raman measurements such as those presented here provide useful insight regarding the progressive accumulation of liquid water at specific locations in the snowpack, e.g., within snow layers that lay on top of a melt-freeze crust. The water content in such layers is critical for a wet avalanche release because the accumulated liquid water reduces the cohesive force among snow grains, thus dramatically decreasing the mechanical stability of the snowpack.

## 2. Results

In Table 1, we report the measured amounts of water accumulated inside the empty container by spraying water from progressively increasing distances from the top open surface. In our experiments, we sprayed water from a point placed between 3 and 5 cm above the container opening (see Section 4 for details). In such a condition, the average collected water per spray is 0.15 g.

We performed four different experiments in which we added controlled amounts of liquid water to different snow batches, as summarized in Table 2, where the conditions adopted to charge with water the two different kinds of snow, namely natural-like snow (NLS) and natural snow (NS) (see Section 4 for details) are grouped. We remark that the density of NLS (280 kg m^−3^) differs from that of NS (380 kg m^−3^). Lower density snow is characterized by a more open microstructure with more pores that result in increased water vapor presence. Around 0 °C enhanced melting is likely to occur. Yet, in our experiments, we discard the role of water vapor due to the dominant weight of the amount of liquid water we deliberately inject in the sample from its top surface.

**Figure 1:**
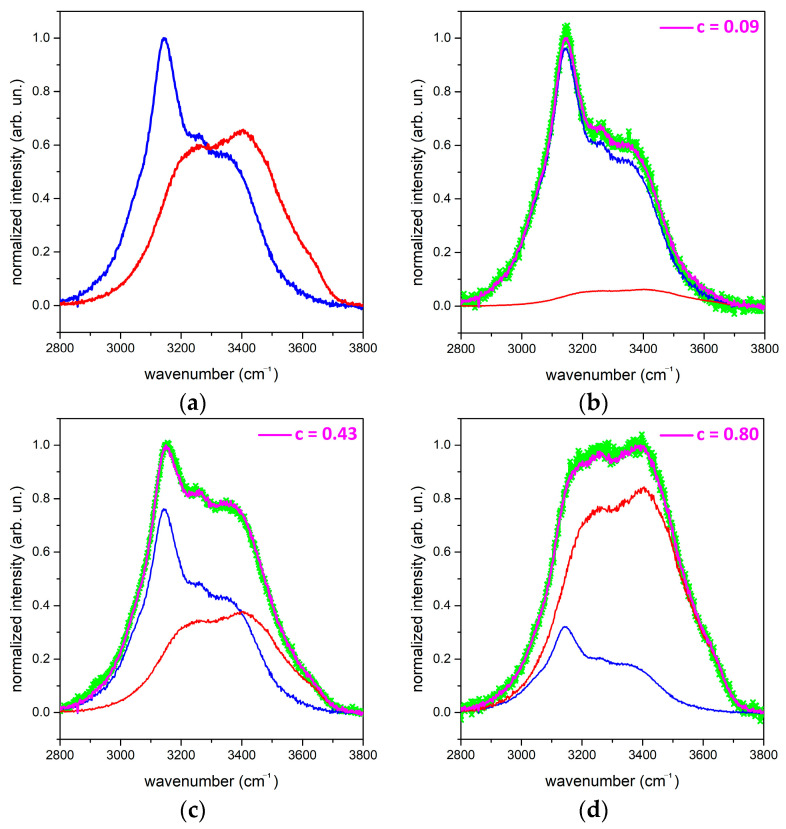
(**a**) RS of ice (blue curve) and liquid water (red curve) at 0 °C normalized to the maximum intensity of the ice spectrum. Panels (**b**–**d**) are the fits (pink curves) to three representative experimental spectra (green curves), as obtained by Equation (1) with the indicated values of the *c* coefficient. In each panel, we report the different relative intensities of the spectra of ice and water.

We collected the reference spectra of bulk ice and liquid water at 0 °C in a mixture of ice/water from a volume of distilled water, focusing the laser spot at the *same* fixed distance for both measurements. We normalized the intensity of both spectra according to the intensity of the most relevant ice peak. The obtained spectra are reported in Figure 1a and clearly show how the different hydrogen-bonding networks that characterize ice and liquid water produce markedly different Raman bandshapes in the OH-stretching region [25]. Such a different spectral profile can be used to *qualitatively* assess, by a least-square fitting procedure, the relative amount of liquid water in snow, as described below.

After the acquisition of the RS of snow, we first operated spike removal and baseline correction over the range 2800–3800 cm^−1^ (OH-stretching band), and the spectra were normalized. To fit all RS collected from snow samples, we use a linear combination of the ice and water spectra according to:

*I_fit_*(*ω*) = (1 − *c*) × *I_ice_*(*ω*) + *c* × *I_water_*(*ω*), (1)

where *I_ice_*(*ω*) and *I_water_*(*ω*) are the intensities of the reference spectra of ice (normalized to its maximum) and water (normalized to the maximum of ice spectrum), respectively. *c* is the fitting coefficient that runs between 0 (pure ice) and 1 (pure liquid water). For a given snow sample, we obtain the value by minimizing the squared sum of the residuals (*S*^2^) computed using the *measured* normalized spectrum of wet snow (*I_meas_*(*ω*)) and the *fitted* spectrum given by Equation (1) in the spectral range of the OH-stretching region (2800 cm^−1^ = *ω_a_* < *ω* < *ω_b_* = 3800 cm^−1^), according to:

(2) $$S^{2}=\int_{\omega_{a}}^{\omega_{b}}{\left[I_{meas}\left(\omega \right)-I_{fit}\left(\omega \right)\right]}^{2}d\omega$$

In Figure 1 b–d, we show the experimental spectra of wet snow samples containing different amounts of water, fitted with the spectrum associated with the value of the *c* coefficient (Equations (1) and (2)) that allows the experimental data to match in the best way (i.e., in the least-squares sense).

For this set of experiments (see Section 4 for details), we used the spray bottle, and we collected six RS after each spray in order to establish a stationary distribution of liquid water throughout the snow volume along the time required to take RS. When we used the pipette, we collected a single RS for every addition of water. Since in this experiment the snow volume is smaller, we assumed that water requires less time to obtain a stationary volume distribution with respect to water addition using the spray bottle.

**Figure 2:**
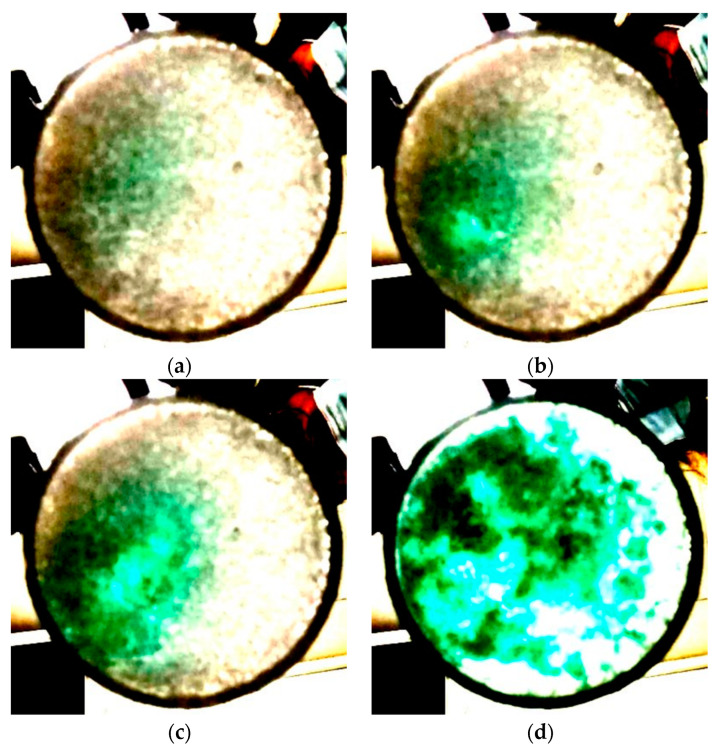
Snow pictures taken from the bottom of the container after uniformly dropping (**a**) 4, (**b**) 7, (**c**) 14, (**d**) 62 pipettes of green-colored water onto the snow top surface. The contrast of the pictures is enhanced to highlight the green-colored areas.

We investigated the percolation of water through the snow by a specific experiment. In Figure 2, we report four representative frames of a video collected during the progressive addition of a diluted (0.03% in volume; see Section 4) water-colored solution to a sample of NS. Moving from plate (a) to plate (d) of the figure, we notice that the fraction of the green-colored area and the intensity of the coloration progressively increase. This coincides with a correspondingly large number of times the fixed liquid water volume (0.04 mL) was uniformly dropped onto the snow surface using the pipette, from 4 (20 s; plate a) to 7 (35 s; plate b) and 14 (70 s; plate c) to 62 (310 s; plate d). We observed that initially (plate a) liquid water was mostly deposited at the top surface of the snow, and it appears as a barely visible green shaded area. The increased intensity of green coloration of plate b coincides with liquid water penetrating down to the bottom of the container through a percolation path. From this point on, water diffusion through snow mostly occurs along the already established path involving progressively larger snow volumes (plate c). After the heaviest water injection, the whole bottom of the container is saturated with liquid water (plate d). The above trend is the same observed in the dye infiltration experiment through snow [26].

**Figure 3:**
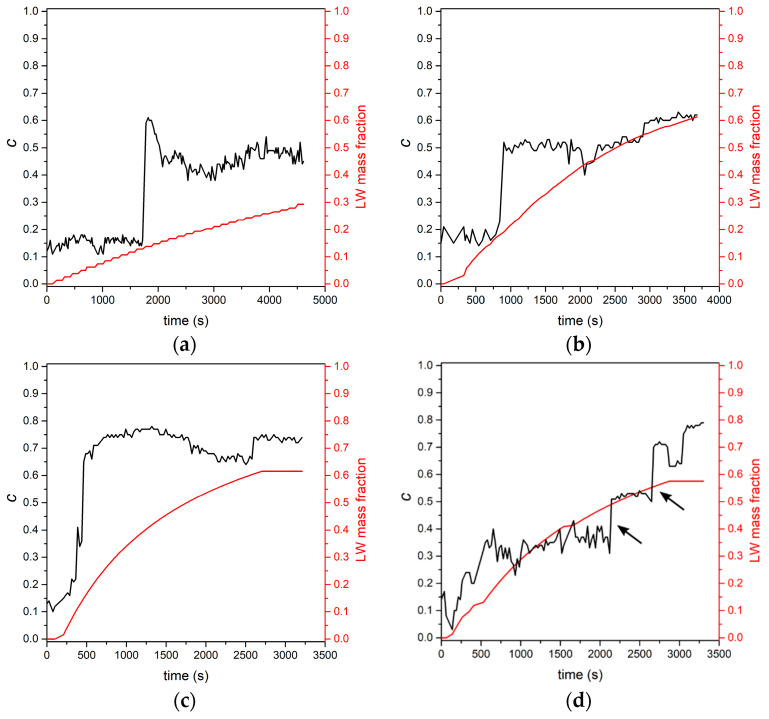
Trend of the *c* coefficient (black curves and ordinate scale) and liquid water mass fraction (red curves and ordinate scale) for selected combinations of snow kind and degree of liquid water injection. (**a**) NLS, spray; (**b**) NLS, pipette; (**c**) NS, pipette 1st; (**d**) NS, pipette 2nd. See text for details.

In Figure 3, we show the trend of the *c* coefficient (Equation (1)) vs. time, as represented by the black curves. The red curves provide the liquid water (LW) mass fraction in the samples. Figure 3a refer to NLS that underwent successive water sprayings. After an initial region where *c* values are nearly constant, a sudden vertical discontinuity (disc. 1) at an LW mass fraction value of 0.13 is followed by a region of roughly constant *c* values.

In Figure 3b, we show the result of an experiment similar to the one discussed in Figure 3a, apart from the more consistent water injection in the snow that was possible when we used a pipette. Indeed, in this case, the mass of the snow sample is about 25% of the sample mass used for spraying measurements (see Table 2). The initial trend of the *c* coefficient is similar to that in Figure 3a: the average *c* value in the initial region is around 0.15. The vertical discontinuity (disc. 1) occurs at the value of LW mass fraction 0.17, and again a nearly flat region of *c* values follows up to a second vertical discontinuity (disc. 2) at the LW mass fraction 0.55. Discontinuity 2 is less marked than discontinuity 1, and it is followed by a region of nearly constant *c* values.

**Figure 4:**
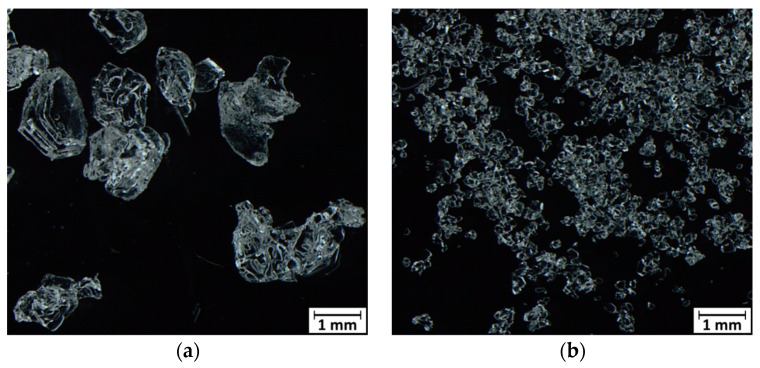
Stereo-microscopy pictures of (**a**) natural and (**b**) natural-like snow grains.

The same kind of measurement just discussed, when performed on a NS sample (Figure 3c), qualitatively mirrors the trend of the *c* coefficient already discussed for Figure 3b. The discontinuities occur at LW mass fraction values of 0.13 and 0.6, respectively. Remarkably, in Figure 3c, the steep increase of the *c* value across the discontinuity region is rather high, around 0.7. We believe that such a large value is due to the combination of the coarse snow grain of this sample (see Figure 4a) and of the focusing conditions. Indeed, since the laser spot diameter is less than the average grain size, it is likely that a small number of snow grains is illuminated, thus magnifying the liquid water contribution in the recorded RS.

In Figure 3d, we show the results we obtained when the same measurement as for Figure 3c was performed under defocused laser irradiation. The consequence is a noisier *c* trend. We identify again the two discontinuities placed at LW mass fractions of 0.13 (disc. 1) and 0.55 (disc. 2), respectively. In between the discontinuities, we observe a roughly constant *c* trend centered around 0.3. Notably, disc 1 is less marked than in all other experiments. Our choice for locating the position of such a discontinuity was driven by the observation that there is a jump in *c* values coincident with an LW mass fraction equal to the value evident in Figure 3c. Two discontinuities occur at high LW mass fraction, as indicated by the arrows in Figure 3d: it is, however, irrelevant which one is considered since the LW mass fraction value for both discontinuities is nearly the same (0.48; 0.51).

In Table 3, we display the LW mass fraction values for our samples at discontinuities 1 and 2.

## 3. Discussion

Some remarks on the results of the evolution of the LW mass fraction in our samples are pertinent. We observe that the initial *c* value differs from 0 in all measurements. This means that even when no liquid water is introduced in the sample, the RS cannot be fitted with the only contribution of bulk ice. We attribute this fact to the role of surfaces in the RS of snow [27] that are covered by a quasi-liquid water layer, the thickness of which becomes relevant over the temperature range −1–0 °C. Such a layer provides liquid water to the RS of snow. Since NLS is made of grains of smaller average size, the contribution of liquid water is larger than in NS. This corresponds to a higher *c* coefficient (about 0.15) for NLS than for NS (on average, 0.1).

Discontinuity 1 observed in Figure 3a–d can be related to the pictures in Figure 2 and to the kinetics of liquid water infiltration through the snow. In the initial stage, the LW content in the volume where the RS is collected is nearly constant. The discontinuity coincides with the time when the liquid waterfront reaches the collection volume of the RS. Then the water level remains nearly constant until water expands through the entire snow volume. We associate the different values taken by the *c* coefficient along this stage in NLS (about 0.5; see Figure 3b) and in NS (about 0.3; see Figure 3d) to the different average grain sizes of the two kinds of snow that behave in a sponge-like manner. In fact, NLS with smaller grains is a more efficient sponge (larger *c*) able to retain a larger amount of liquid water than NS. The above *c* values are in agreement with the volumetric water content measured with other techniques [28].

Discontinuity 2 is associated with the formation of a layer completely filled with liquid water at the bottom of the sample, as we observed. In this condition, since the laser is focused at a fixed height with respect to the sample bottom, the fractional contribution of liquid water to RS, with respect to that of ice, becomes dominant.

The experiments discussed above appear suitable to detect the presence of liquid water in a snow volume and to follow the evolution of the progressive filling of snow with the liquid phase. This is facilitated by the spectral shape analysis of the RS of the wet snow sample, based on the weighted contributions of the spectral features of ice and water. Indeed, the recorded RS result solely from the vibrational contributions of water and ice. The contributions from other species, such as chemical contaminants and dust, provide additional peaks that can be easily spotted and rejected in the analysis of the RS. Thus, Raman spectroscopy opens the way to the investigation of the spatial-temporal changes in the water content of snow using a non-destructive and minimally invasive optical approach. For on-field applications, a vertical trench across the snowpack thickness is required to take RS at different depths corresponding to progressively accumulated snow layers. For a quantitative estimate of θ_w_, we still have to determine the calibration curve of our method against θ_w_, as measured by an assessed, independent technique. We plan to exploit this activity in future research.

## 4. Materials and Methods

We performed our experiments inside a home-built climatic chamber. We recorded the temperature by placing a type K thermocouple at the bottom (immersed in stagnant air) of a commercial freezer. We connected the thermocouple to an Arduino board (model Uno) feedback looped to a relay that acts as a switch and turns on the freezer when the temperature recorded by the thermometer is higher than −1 °C. With this system, we kept the temperature inside the climatic chamber between −2 °C and −0.5 °C. The top opening of the freezer was closed with a 2 cm thick Styrofoam plate; we cut a circular opening at the center of the plate, through which we could inject water on the top surface of the sample. Given the low thermal conductivity of air, we assume that over the temperature range explored during our experiments, neither melting of snow nor freezing of water occurred in association with heat exchanges between the snow samples and the surrounding air.

To reduce, as much as possible, the melting of snow grains during the addition of liquid water, we used distilled water at 0 °C to avoid handling of undercooled water with the associated metastability. We partially frost a volume of water to constantly keep liquid water at 0 °C during the experiments.

We filled a cylindrical Polypropylene container with an external diameter of 39 mm, a height of 6 cm and 2 mm thick lateral surfaces with snow, without compacting it. We removed the bottom of the container, and we closed it with an optically transparent Polyethylene (PE) film (thickness 10 µm) impermeable to liquid water. The film was stretched to obtain folding-free transparent sealing to ensure optimal quality of the recorded RS.

We added liquid water to the snow using a spray bottle and a pipette. Our purpose is to simulate the random fall of raindrops on the snowpack surface.

In the first set of experiments, we used a spray bottle that produced a puff of water droplets of average size between 200 µm and 600 µm. The water mass ejected in each spray was about 0.22 g. The amount of liquid water collected in the container was smaller than the injected water mass because a fraction of the droplets evaporate or fall outside the container. In the second set of experiments, we added liquid water to snow using an adjustable volume pipette (Model: Gilson P1000). The pipette was kept 2 cm above the top open surface of the snow. Under these conditions, the whole ejected water fell onto the snow surface. In all pipette experiments, we set a fixed volume of 0.04 mL per injection.

We used two reference types of snow: natural snow (NS) and natural-like snow (NLS). NS was collected from a field at Gressoney Sant Jean (Aosta Valley, Italy). It was gently collected using a plastic scoop and filled in plastic containers avoiding any compaction. We stored NS inside the closed containers at a temperature of −15 °C for seven months. After this period, the average size (taken out of 20 randomly chosen grains) of NS was 1.6 mm, as shown in Figure 4a, and the density of NS was 380 kg m^−3^.

We produced NLS using the method described in [29]. NLS was stored under the same temperature conditions as NS for 2 months after synthesis. Due to metamorphism, the average snow grain size increased up to 0.2 mm (taken out of 20 different snow grains), as shown in Figure 4b. The density of NLS was 280 kg m^−3^.

The pictures of the snow samples were acquired with an Olympus SZX16 stereomicroscope(Tokyo, Japan) connected to an LC30 Olympus digital color camera.

To visually look at the process of water spreading through the two kinds of snow, we colored a sample of liquid water with a diluted solution (0.03% in volume) of a green dye. The color of the solution is transparent greenish. We maintained the solution at 0 °C. We placed 3 g of natural snow inside the cylindrical container, and using the pipette, we carried out successive additions of 0.04 mL each of the colored solution every 5 s. Simultaneously, we acquired a video by using a smartphone to monitor the evolution of the snow-solution mixture at the bottom of the container.

RS were collected with portable Raman equipment BW&TEK (Plainsboro, NJ, USA) Exemplar Plus model (wavelength range 532–680 nm; slit width 10 µm, diffraction grating 1800 lines mm^−1^, resolution 2 cm^−1^). All spectra were acquired at the fixed laser wavelength 532 nm and laser power of 50 mW, with an acquisition time of 15 s. We used a BAC102 Raman probe (BW&TEK, Plainsboro, NJ, USA) that operates in backscattering. The focal plane is located at a distance of 5.4 mm from the probe. The diameter of the laser spot at the focal plane is 85 µm.

**Figure 5:**
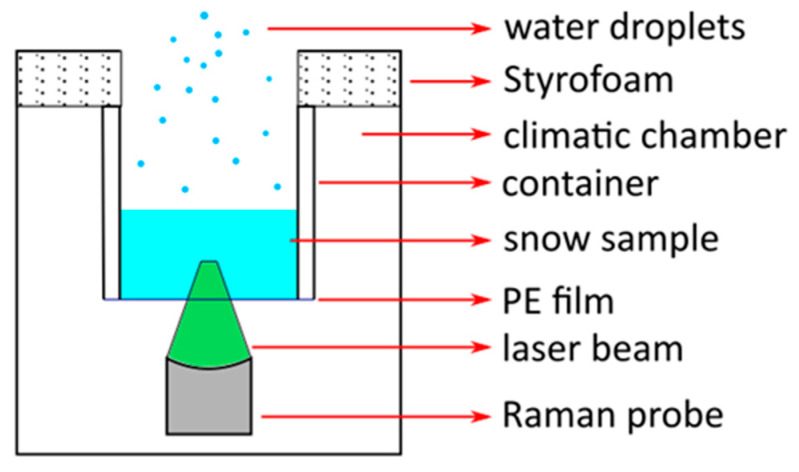
Schematic of the Raman probe disposition with respect to the snow sample. The green triangular area represents the laser focus position within the snow volume.

The Raman probe was placed upward toward the center of the circular bottom side of the container closed with the PE film (Figure 5). We adjusted the probe-container distance in such a way that the focal plane was located within the snow volume far enough from the PE film to facilitate negligible PE contributions to the RS. We estimate that the diameter of the laser spot at the internal surface of PE film was between 0.2 and 0.4 mm.

## 5. Conclusions

In conclusion, we proved that Raman spectroscopy is a powerful technique to assess the presence of liquid phase in snow. We performed a demonstrative study analyzing in the laboratory qualitatively different kinds of snow, representative of the two extremes of freshly deposited, fine-grained snow characteristic of early winter snowfalls (NLS) and of coarse-grained snow typical of aged layers in a snowpack (NS). We observed two definite discontinuities in the trend of the *c* coefficient vs. time. These are associated with different degrees of snow impregnation with water. The percolation kinetics are different in the two kinds of snow. Raman spectroscopy is appreciable because the instrumentation is light, easy to carry on the field and affordable. The measurements are fast and scarcely invasive. By data analysis procedures, the collected spectra can be processed to easily remove spectral contributions from impurities accidentally present in the snowpack.

## Figures and Tables

**Table 1:** Water mass collected in the container after spraying from progressively increased heights above the top open surface.

Height (cm)	Collected Water after 100 Sprays (g)	Average Collected Water per Spray (g)
0	18.3	0.183
5	11.6	0.116
10	3.9	0.039
15	3.3	0.033
20	2.6	0.026

**Table 2:** Experimental parameters adopted to add liquid water to the snow samples.

Snow Kind; Injection Method	Initial Snow Mass (g)	Total Mass of Injected Water (g)	Number of Injection Steps	Water Mass per Unit Step (g)
NLS; spray	11.6	4.8	31	0.15
NLS; pipette	3	4.7	100	0.047
NS; pipette 1st	3	4.8	100	0.048
NS; pipette 2nd	3.1	4.2	100	0.042

**Table 3:** LW mass fraction at the *c* discontinuities.

Snow Kind; Injection Methos	LW Mass Fraction at Discontinuity 1	LW Mass Fraction at Discontinuity 2
NLS; spray	0.13	
NLS; pipette	0.17	0.55
NS; pipette 1st	0.13	0.55
NS; pipette 2nd	0.13	0.6

## Data Availability

The data presented in this study are available on request from the corresponding author.

## Sample Availability

Samples of the compounds are not available from the authors.
